# Pathogenesis of Target Organ Damage in Hypertension: Role of Mitochondrial Oxidative Stress

**DOI:** 10.3390/ijms16010823

**Published:** 2014-12-31

**Authors:** Speranza Rubattu, Beniamino Pagliaro, Giorgia Pierelli, Caterina Santolamazza, Sara Di Castro, Silvia Mennuni, Massimo Volpe

**Affiliations:** 1 Department of Clinical and Molecular Medicine, School of Medicine and Psychology, University Sapienza of Rome, Ospedale S. Andrea, Rome 00189, Italy; E-Mails: beniamino.pagliaro@libero.it (B.P.); giorgia.pierelli@alice.it (G.P.); caterina.santolamazza@gmail.com (C.S.); silvia.mennuni@gmail.com (S.M.); massimo.volpe@uniroma1.it (M.V.); 2 Istituto di Ricovero e Cura a Carattere Scientifico (IRCCS) Neuromed, Pozzilli 86077, Italy; E-Mail: saradicastro@yahoo.it

**Keywords:** target organ damage, hypertension, mitochondrial dysfunction, oxidative stress

## Abstract

Hypertension causes target organ damage (TOD) that involves vasculature, heart, brain and kidneys. Complex biochemical, hormonal and hemodynamic mechanisms are involved in the pathogenesis of TOD. Common to all these processes is an increased bioavailability of reactive oxygen species (ROS). Both *in vitro* and *in vivo* studies explored the role of mitochondrial oxidative stress as a mechanism involved in the pathogenesis of TOD in hypertension, especially focusing on atherosclerosis, heart disease, renal failure, cerebrovascular disease. Both dysfunction of mitochondrial proteins, such as uncoupling protein-2 (UCP2), superoxide dismutase (SOD) 2, peroxisome proliferator-activated receptor γ coactivator 1-α (PGC-1α), calcium channels, and the interaction between mitochondria and other sources of ROS, such as NADPH oxidase, play an important role in the development of endothelial dysfunction, cardiac hypertrophy, renal and cerebral damage in hypertension. Commonly used anti-hypertensive drugs have shown protective effects against mitochondrial-dependent oxidative stress. Notably, few mitochondrial proteins can be considered therapeutic targets on their own. In fact, antioxidant therapies specifically targeted at mitochondria represent promising strategies to reduce mitochondrial dysfunction and related hypertensive TOD. In the present article, we discuss the role of mitochondrial oxidative stress as a contributing factor to hypertensive TOD development. We also provide an overview of mitochondria-based treatment strategies that may reveal useful to prevent TOD and reduce its progression.

## 1. Introduction

Arterial hypertension represents a major cardiovascular epidemic condition in both the developed and developing world, which remains asymptomatic until late in its course [1]. Hypertension has a prevalence of 26.4% in the adult population, totaling nearly one billion individuals [2]. Its prevalence has been estimated to increase up to 29% (1.5 billion) by the year 2025.

As a consequence of hypertension, specific organs suffer with resulting cardiac disease, cerebrovascular disease and vascular dementia, renal failure, atherosclerotic vascular disease, and retinopathy—Hence the term “target organ damage” (TOD) [3]. Hypertension more than doubles the risk of coronary heart disease and triples the risk of heart failure and stroke [4].

Although elevated blood pressure *per se* is undoubtedly the major factor contributing to hypertensive TOD, there is clear evidence that other mediators are also crucially involved. This observation raises the issue of a multifactorial approach aimed not only at achieving target blood pressure levels, but also at preventing the development and progression of TOD in order to reduce the overall cardiovascular risk of patients [5,6].

## 2. Molecular Mechanisms of Target Organ Damage (TOD): Focus on Oxidative Stress and Mitochondria

Several processes are involved in the pathogenesis of TOD and these include endothelial activation, platelet activation, changes of collagen turnover, of coagulation and fibrinolytic pathways, and of matrix metalloproteinases (MMPs) [7]. Moreover, development and progression of TOD may be amplified by arterial stiffness [8,9], the amplitude of concomitant heart rate changes [10], the effects of sympathetic overactivity [11], the remodeling of extracellular matrix (ECM) [12,13], as well as by several hormonal systems including the renin-angiotensin-aldosterone system (RAAS).

Interestingly, common to all these processes is an increased bioavailability of reactive oxygen species (ROS) known as oxidative stress. ROS behave as an important intracellular and intercellular second messenger that modulate many downstream signaling molecules leading to vascular smooth muscle cell (VSMC) growth and migration, expression of proinflammatory mediators and remodeling of ECM. In addition, ROS increase intracellular free calcium concentration, a major determinant of vascular reactivity [14,15].

The most relevant sources of ROS with respect to vascular disease and hypertension are:

1. Nicotinamide adenine dinucleotide phosphate (NADPH) oxidase;
2. Uncoupled nitric oxide synthase (NOS);
3. Xanthine oxidase;
4. Mitochondria.

It is known that there is an important feed-forward interplay between these ROS sources [16,17].

Mitochondria mediate oxidative phosphorylation (OXPHOS) via electron transfer through multimeric complexes to produce ATP [18]. ROS are produced as a byproduct of the respiratory chain, making mitochondria the major source of cellular ROS [19].

Within mitochondria, the Nox4 subunit of NADPH oxidase is also involved in producing anion superoxide (O_2_^•−^) and it plays a central role in cardiovascular damage. Nox4 is localized in mitochondria of various cell types, including cardiomyocytes, renal mesangial cells and neuronal cells [20]. In particular, the latter study has demonstrated the Nox4 localization to neuronal mitochondria, suggesting a causal link between Nox4 expression and mitochondrial O_2_^•−^ production upon Ang-II stimulation [20]. Moreover, Nox4 up-regulation has been shown to cause increased mitochondrial O_2_^•−^ production in response to pressure overload in cardiac myocytes, contributing to apoptosis [21]. Interestingly, mitochondrial Nox4, apart from producing ROS, can be itself modulated by ROS levels in the diabetic kidney [22].

Although the role of mitochondrial ROS is not completely understood, it is proposed that mitochondrial dysfunction causing excessive ROS production may be a prominent feature of most cardiovascular diseases (CVDs) [23]. Mitochondria may be involved in the genesis of hypertension [24] and hypertension itself may promote mitochondrial dysfunction in the brain, heart, vasculature and kidneys [25]. Since these organs are involved in the development of hypertension, it is likely that mitochondrial dysfunction may contribute to both sustaining hypertension as well as to promoting TOD [24,25].

This review will focus on the role of mitochondrial oxidative stress as a contributing factor in the development of TOD in hypertension.

## 3. Mitochondrial Dysfunction and Vascular Hypertensive Damage

Damage to the endothelium is considered the initiating step of atherosclerosis [26], with low density lipoprotein (LDL) uptake and leucocyte adhesion and migration occurring at sites of endothelial dysfunction [27].

Both *in vitro* and *in vivo* studies outlined the importance of mitochondrial dysfunction, as a source of increased ROS, in the pathogenesis of endothelial dysfunction and, consequently, of atherosclerosis [23]. Increased ROS production in mitochondria leads to lipid, protein, and mtDNA damage. The latter is the most sensitive molecule to physiologically relevant ROS-mediated insult. VSMCs and endothelial cells (ECs) exposed to ROS show a preferential increase in mtDNA damage, in addition to decreases in steady-state levels of mtDNA-encoded mRNA transcripts, mitochondrial protein synthesis, and membrane potential, consequently decreasing total cellular ATP pools [28].

Studies performed in experimental models of Angiotensin II (Ang II)-infused mice and in ECs exposed to Ang II [29] support a link between both cytoplasmic and mitochondrial ROS with endothelial dysfunction in hypertension [30]. In fact, bovine aortic ECs exposed to Ang II showed mitochondrial dysfunction with increased mitochondrial ROS production and decreased membrane potential, decreased respiratory control ratio and low molecular weight thiols content [31]. These deleterious effects of Ang II on mitochondrial function were associated with increased cellular O_2_^•−^ production and decreased endothelial nitric oxide (NO) bioavailability.

Ang II-mediated mitochondrial dysfunction is achieved through binding with Ang II type 1 (AT1) receptor (a Gαq-coupled receptor) and activation of vascular NADPH oxidase in a protein kinase C (PKC)-dependent manner. Notably, ROS derived from NADPH oxidase may increase mitochondrial ROS production. Inhibition of both NADPH oxidase and PKC by apocynin and chelerythrine, respectively, completely prevented mitochondrial dysfunction induced by Ang II [31,32].

In addition, the close relationship between non-mitochondrial and mitochondrial ROS in the pathogenesis of endothelial dysfunction was investigated in cultured human aortic ECs. In fact, it was shown that the Nox2 isoform of vascular NADPH oxidase is responsible for Ang II-mediated stimulation of mitochondrial ROS in human ECs. Depletion of Nox2 inhibited Ang II-induced production of mitochondrial O_2_^•−^. Moreover, the interplay between mitochondrial and NADPH oxidase-derived O_2_^•−^ constitutes a feed-forward cycle in which Nox2 increases the production of mitochondrial ROS by reverse electron transfer [33].

Among the mitochondrial proteins underlying ROS-induced vascular dysfunction in hypertension, the aldehyde dehydrogenase (ALDH2) appears to be one of the most interesting. This enzyme detoxifies aldehydes to carboxylic acids and may be considered as a novel antioxidant target to improve vascular function. Its inhibition in the model of Ang II hypertensive mice [29] was related to increased oxidative stress and to ROS-induced vascular contraction contributing to vascular dysfunction and structural remodeling in hypertension. An additional mitochondrial protein involved in ROS-induced vascular dysfunction is uncoupling protein-2 (UCP2), located within the inner mitochondrial membrane, and highly involved in the regulation of vascular structure and function. The protective role of UCP2 overexpression towards salt-induced vascular dysfunction was shown in transgenic mice specifically overexpressing human UCP2 in VSMCs [34]. Furthermore, UCP2 overexpression preserved endothelial function in diet-induced obese mice [35]. These studies clearly showed that the beneficial effects of UCP2 overexpression in the vasculature was associated with a decreased production of O_2_^•−^ and with an increased NO bioavailability. Recently, the role of UCP2 in mediating the endothelial protective effects conferred by a dipeptidyl-4 inhibitor (sitagliptin) in hypertension was reported [36].

Finally, evidence obtained in humans highlights a defect of antioxidants level (vitamin C) with a concomitant increase of inflammation and of atherosclerosis in patients with peripheral artery disease, also including hypertensive patients [37].

## 4. Mitochondrial Dysfunction and Cardiac Hypertensive Damage

Cardiac hypertrophy is a well-known compensatory response to increased workload as an adaptation to changes in wall stress so as to maintain cardiac output in hypertension [38]. Transition from a physiological to a pathological condition is associated with a series of changes in cardiac metabolism that ultimately lead to a dysfunctional phenotype [39]. Briefly, fatty acid oxidation, a primary energy source in normal conditions, takes place in pathological hypertrophy switching to non-mitochondrial metabolism (glicolysis). In turn, glucose oxidation may be reduced once heart failure (HF) develops, ultimately leading to ATP production impairment [40].

Molecular signaling pathways linking ROS to cardiac hypertrophy and remodeling in hypertension include calcium channels (ICa), α and β adrenergic receptors, Ang II AT1 receptor, collagen and MMPs, modification of stress kinases (mitogen-activated protein kinases-MAPK), phosphoinositol 3-kinase (PI3K), apoptosis signaling kinase-1 (Ask-1), PKC, sarcomeric and excitation–contraction coupling proteins, nuclear transcription factors such as nuclear factor κB, activator protein-1, peroxisome proliferator-activated receptor γ coactivator 1-α (PGC-1α) [41,42].

The majority of data derive from studies exploring classical molecular pathways of ROS production, *i.e.*, activation of NADPH oxidase [43,44], of xanthine oxidase [45] and uncoupling of NO synthase, especially endothelial NOS or NOS3 [46]. With regard to the role of mitochondria, it has been suggested that increased ROS levels would occur at the extremes of overall intracellular and intra-mitochondrial redox potential, which in turn depends on redox couples involved in both ROS generation (NADH/NAD^+^) and ROS scavenging (NADPH/NADP^+^) [47]. An increase in ROS generation occurs when mitochondrial redox potential is significantly reduced, as it happens in hypoxia condition, or when it is significantly oxidized, as it may happen during HF [48].

The pressure overload-induced HF represents a suitable experimental model of cardiac hypertensive damage in humans. In this model, induced by aortic arch constriction applied for different time periods in Sprague–Dawley rats, the pressure overload-induced impairment in fatty acid oxidation preceded onset of congestive HF. It is noteworthy that mitochondrial respiratory capacity was maintained until the ejection fraction decreased. These temporal relationships suggested a tight link between impaired substrate oxidation capacity and contractile dysfunction in HF development [49].

Interestingly, the application of a proteomic approach in this animal model led to the discovery of a down-regulation of fatty acid oxidation enzymes, of an up-regulation of pyruvate dehydrogenase subunits and of several bidirectional changes in ETC subunit composition. This “remodeling” of the mitochondrial proteome was associated with severe defects in mitochondrial and whole heart oxidative capacity, supporting the concept that a global oxidative defect due to mitochondrial dysfunction contributes to the onset and/or severity of cardiac dysfunction following transition of pressure overload hypertrophy to overt HF [50]. Interestingly, when a band is placed around the aorta to induce pressure overload in mice lacking PGC-1α they develop signatures of HF, including a marked drop in cellular ATP concentration. This evidence strongly suggests that mitochondrial derangements known to occur in the failing heart of this model are, at least in part, related to a down-regulation of PGC-1α regulatory cascade [42].

The link between mitochondrial calcium and oxidative stress in promoting cardiac damage has been investigated. Elevated sodium concentration in cardiomyocytes of the failing heart reduced mitochondrial calcium by accelerating its efflux and by decreasing NADPH oxidase expression level, therefore resulting in increased mitochondrial ROS formation. On the other hand, a decrease in calcium during increased workload reduced the mitochondrial antioxidant capacity by decreasing the activity of Krebs cycle dehydrogenases [51].

Induction of the radical defense plays a key role in protecting the failing myocardium, as shown in SHR models that are resistant to heart damage induced by H_2_O_2_ as a result of elevated activities of glutathione peroxidase and superoxide dismutase (SOD) [52]. In addition, Dai *et al.*, used genetic mouse models overexpressing catalase targeted to mitochondria and to peroxisomes (cytoplasmic). Mice overexpressing catalase targeted to mitochondria, but not mice overexpressing cytoplasmic catalase, are resistant to cardiac hypertrophy, fibrosis and mitochondrial damage induced by Ang II, as well as to HF induced by overexpression of Gαq. Furthermore, primary damage to mtDNA was shown to contribute directly to development of cardiac hypertrophy, fibrosis and failure [53]. Overall, these data underscore the critical role of mitochondrial ROS in cardiac hypertrophy and failure and strongly support the potential use of mitochondrial-targeted antioxidants for prevention and treatment of hypertensive cardiomyopathy [54,55], as discussed in the therapeutic paragraph below.

## 5. Mitochondrial Dysfunction and Renal Hypertensive Damage

Arterial hypertension is a leading cause of end-stage renal disease and one of the main factor responsible for progression of glomerular and tubulo-interstitial diseases [56].

Recent data indicate that oxidative stress is a pivotal molecular mechanism involved in the pathogenesis of hypertensive renal damage [57].

The kidney is a highly energetic organ and it is rich in mitochondria [58]. Thus, mitochondrial dysfunction plays a critical role in the pathogenesis of kidney diseases [59]. The important difference in oxygen supply between the renal cortex and the medulla is related to differences in the metabolism and different susceptibility to injury of the two renal regions. Oxidative metabolism, mostly from fat, is the major source of energy for cortical proximal tubular cells. The enzymes responsible for gluconeogenesis are limited to the proximal tubule. The enzymes involved in glycolysis, on the other hand, predominate in distal structures of the nephron [58].

O_2_^•−^ has been identified as the most important ROS involved in vascular and tubular dysfunction in hypertension. H_2_O_2_ reduces preglomerular vascular reactivity by promoting medullary blood flow and pressure natriuresis in hypertensive animals. Increased renal ROS are involved in renal vasoconstriction, renin release, activation of renal afferent nerves, augmented contraction, and myogenic responses of afferent arterioles, enhanced tubuloglomerular feedback, dysfunction of glomerular cells, and proteinuria [60].

In SHR, a decline of kidney mitochondrial function is documented by increased H_2_O_2_ generation and reduced UCP2 expression, associated with decreased cytocrome oxidase, Mn-SOD and NOS activities, leading to impaired membrane mitochondrial potential [61]. These alterations were prevented by treatment with an Ang II AT1-receptor blocker, but not with a calcium-antagonist, further stressing the key role of Ang II in promoting mitochondrial dysfunction in hypertension [61]. Moreover, the pressure-induced ROS formation appeared related to Ang II rather than to norepinephrine levels [62].

Other animal models support the link between mitochondrial function and hypertensive renal damage. In the model of Dahl/Rapp salt-sensitive rat, high-salt dietary feeding led to histological lesions similar to human nephrosclerosis. This damage was associated with mitochondrial dysfunction and with abnormal release of cytocrome c and increased apoptosis [63].

The high-salt fed SHRsp rat develops severe degree of renal damage compared with stroke-resistant SHR, despite comparable blood pressure levels [64]. This model shows a marked down-regulation of UCP2 gene expression in the kidneys and a significant increase of tissue inflammation, oxidative stress and histological damage [64]. Consistently, UCP2 plays a key role in protection from oxidative stress-induced damage in renal mesangial cells *in vitro* [64].

Mitochondrial dysfunction appears to be associated with an increased occurrence of renal hypertensive damage also in mice. In fact, high-salt diet resulted in hypertension with an increased degree of oxidative stress and of inflammation in the kidneys of mitochondrial SOD-deficient (MnSOD^−/−^) mice, but not in the kidneys of wild-type mice [65].

## 6. Mitochondrial Dysfunction and Cerebral Hypertensive Damage

Arterial hypertension, cerebrovascular disease and dementia are strictly associated. Stroke is the leading cause of adult disability.

Alterations of energy production that occur in cerebral cells represent the basis for several brain disorders. Brain mitochondria produce about 90% of the energy used by cells [66]. The energy is needed for communication among brain cells, which is crucial for transmitting stimuli and signals and thus for optimal function. Adequate energy supply by mitochondria is essential for neuronal excitability and survival.

Mitochondria are implicated in the pathogenesis of neurodegenerative diseases and cerebral ischemia. Recent evidence has suggested an intimate link between excessive ROS generation and development of neuronal death in brain disorders [67,68]. In fact, the brain is particularly prone to oxidative damage for several reasons: high calcium traffic across neuronal membranes, presence of excitotoxic amino acids and autoxidizable neurotransmitters, and a high amount of polyunsaturated fatty acids contained within membrane lipids that become the target of oxygen-derived free radicals. Moreover, the brain is relatively deficient in antioxidant defenses [66].

ROS contribute not only to injury of macromolecules, but also to transduction of apoptotic signals. Overproduction of ROS by the respiratory chain of brain mitochondria can progressively impair mitochondrial energy metabolism in hypertension. This phenomenon may be implicated in the vulnerability to cerebral ischemia, resulting in a progressive neuronal cell death [66]. In fact, persistently elevated blood pressure levels can cause metabolic damage and mitochondrial alterations that favor cognitive dysfunction [69].

Experimental evidence linking mitochondrial dysfunction with occurrence of hypertensive cerebrovascular damage is scarce. Interestingly, an assembly defect in I and V mitochondrial complexes was found in SHR brain mitochondria [70]. Complex I, or NADH:ubiquinone oxidoreductase, is the largest of the five OXPHOS complexes [71]. It contributes about 40% of the proton motive force that drives ATP synthesis by ATP synthase [72,73]. Complex V is responsible for the catalytic phosphorylation of ADP to form ATP. Thus, as a consequence of the observed assembly defects, a decreased ATP production and cell energy deficiency was detected in the brain of SHR [70].

It is also known that mitochondria contribute to both intracellular redox status and calcium homeostasis [74]. In this regard, a decreased complex IV activity during age-associated hypertension was related to a reduction of mitochondrial inner-membrane potential and changes in Ca^2+^ movements in SHR with consequent impairment in the activity of Ca^2+^-dependent enzymes like mitochondrial isoform of nitric oxide synthase (mtNOS). These alterations may certainly contribute to a progression of mitochondrial dysfunction and neuronal cell death during hypertension [75]. It is interesting that the mtNOS, located within the inner mitochondrial membrane, is involved in the modulation of transmembrane potential, inhibition of respiration and ATP synthesis, permeability transition pore opening and apoptosis, in addition to being correlated with onset of hypertensive damage [76,77].

## 7. Therapeutic Approaches

It is widely acknowledged that antioxidant approaches exert beneficial effects in CVDs [78] and many efforts have been made to strengthen this evidence. Of interest, among lifestyle changes, caloric restriction was shown to ameliorate Ang II-induced cardiomyocyte hypertrophy, vascular inflammation, cardiac damage and fibrosis, cardiomyocyte apoptosis, and to increase mitochondrial activity and cellular stress resistance [79]. In addition, the utilization of l-carnitine, a co-factor in fatty acid metabolism by mitochondria, was shown to reduce cardiac remodeling in experimental hypertension through an interaction with the mitochondrial respiratory chain [80] (Table 1).

**Table 1:** Therapeutic approaches.

Agents	Therapeutic Effects
**l-Carnitine**	↓ Cardiac remodeling in experimental hypertension [80]
**Conventional antioxidants (Vitamin C, Vitamin E**	↓ MPO activity; ↓ Lipids peroxidation [81,82,83,84,85,86]; ↓ Atherosclerosis process rate
**ACEI, ARB, Statins**	↑ Vascular eNOS activity; ↓ Endothelin-1 expression; ↓ Ang II AT1 receptor expression [87,88,89,90,91,92]; ↓ NADPH oxidase activity
**Mitochondria-targeted antioxidant therapies**	
**Mito Q10**	↓ Lipid peroxidation and mitochondria damage; ↑ Endothelial NO bioavailability [93,94,95,96]; ↓ Cardiac hypertrophy in young SHRSP
**SS-31**	↓ Ang-II induced mitochondrial oxidative stress; ↓ Up-regulation of mitochondrial biogenesis; ↓ ROS mediated p38 MAPK signaling [97,98]; ↓ Caspase-3 activation [54]
**Edaravone**	↓ Pressure overload-induced left ventricular hypertrophy (Ask1 inhibition); ↓ Perivascular and intermuscular fibrosis [99]

Ang: Angiotensin; MPO: Myeloperoxidase; CVDs: Cardiovascular diseases; ACEI: Angiotensin-converting enzyme inhibitor; ARB: Angiotensin II receptor blocker; eNOS: Endothelial nitric oxide synthase; AT1: Angiotensin II receptor type 1; NADPH: Nicotinamide adenine dinucleotide phosphate; NO: Nitric oxide; SHRSP: Spontaneously hypertensive stroke prone rat; ROS: Reactive oxygen species; MAPK: Mitogen-activated protein kinase; Ask1: Apoptosis signal-regulating kinase 1.

However, a large number of trials [81,82,83] have conclusively shown that antioxidants do not exert the expected preventive and therapeutic effects. In this regard, failure of the conventional antioxidants may be mostly related to their inability to reach the mitochondria. In addition, vitamin C administered as a dietary supplement to humans has been shown to exhibit pro-oxidant properties, which may give rise to paradoxical effects in clinical intervention trials [84]. Other reasons for the negative effects observed with vitamin administration may explain vitamin C status, differences in vitamin C intake, and confounding effects of other medical treatments (*i.e*., statins, blood pressure-lowering drugs). Moreover, genotypic variations among individuals for genes controlling vitamin C metabolism and bioactivity may have played a role [85,86].

Several drugs routinely used for CVD treatment have shown protective effects towards oxidative stress-induced TOD in hypertension. For example, the vasculo-protective effects of statins may have important implications for preventing TOD in hypertension by maintaining vascular eNOS activity, reducing endothelin-1 expression, down-regulating Ang II AT1 receptor expression, and by inhibiting NADPH oxidase activity, with the consequent inhibition of oxidative stress accumulation [87,88,89,90].

Both Ang II AT1 receptor blockers (ARB) and Ang I converting enzyme inhibitors (ACEI) are known to protect from renal injury in hypertension, and at least part of this protection appears to be independent of blood pressure reduction [91]. Previous work showed that ARB and ACEI can improve antioxidant status and attenuate oxidative stress [92].

A recent study assessed the effects of ARB (losartan) on mitochondrial function in SHR. Losartan and amlodipine treatments were equally effective in reducing blood pressure, but only losartan prevented mitochondrial dysfunction and attenuated structural and functional changes in the kidneys. Thus, maintenance of the glutathione pool in a relatively more reduced status, preservation of Mn–SOD activity, and attenuation of UCP2 content reduction in losartan-treated, but not in amlodipine-treated, SHR support a link between Ang II inhibition and mitochondria in renal damage protection in hypertension [61].

Antioxidant therapies specifically targeted to mitochondria represent interesting novel molecules able to reduce oxidative stress [100]. In fact, they may be able to overcome the difficulties so far encountered with the use of conventional antioxidants [81,82,83].

A recently developed mitochondria-targeted ubiquinone, MitoQ10, overcomes this problem. MitoQ10 is composed of a lipophilic triphenylphosphonium cation (decylTPP) covalently attached to a ubiquinol antioxidant [93,94] that can easily move through phospholipid bilayers without requiring a specific uptake mechanism [95]. Within mitochondria, MitoQ10 is reduced by the respiratory chain to its active ubiquinol form, which is an effective antioxidant that prevents lipid peroxidation and mitochondria damage. This protective mechanism appears to depend on the accumulation of ubiquinol within the mitochondria, because the control substance, decylTPP, had no beneficial action. MitoQ10 provides a novel approach to attenuate mitochondria-specific oxidative damage with the potential to become a novel therapeutic strategy for the treatment of CVDs. In fact, oral administration of MitoQ10 protected against development of hypertension improved endothelial NO bioavailability, and reduced cardiac hypertrophy in young SHRSP. The attenuation of blood pressure rise in young SHRSP suggested that prevention of mitochondrial oxidative damage at an early age can provide significant hemodynamically beneficial effects [55]. Addition of MitoQ10 to losartan has been recently tested in the SHRSP model with evidence of remarkable protective effects towards TOD development [96].

Other antioxidant molecules specifically targeted to mitochondria have been developed, such as the Szeto-Schiller (SS)-31 and Edaravone.

SS-31 is selectively targeted at the mitochondria inner membrane and can scavenge anion superoxide, hydrogen peroxide, peroxynitrite, and hydroxyl radicals [101,102]. SS-31 significantly attenuated Ang-II-induced mitochondrial oxidative stress, reduced upregulation of mitochondrial biogenesis, and reduced ROS-mediated p38 MAPK signaling. Inhibition of p38 MAPK was shown to improve cardiac remodeling and inflammation and to preserve cardiac function in HF [97,98].

Moreover, several studies showed that SS-31 prevented caspase-3 activation, along with amelioration of Ang II-induced cardiomyopathy. The SS-31 had no effect on blood pressure levels but exerted direct cardioprotective mechanisms involving reduction of cardiac mitochondria damage with amelioration of apoptosis and fibrosis [54].

Edaravone was shown to exert an inhibitory effect on a water-soluble and a lipid-soluble peroxyl radical–induced peroxidation system and to have sufficient accessibility to tissues, including heart, so that it can effectively scavenge tissue ROS [103]. In *in vivo* murine model, treatment with edaravone was shown to significantly attenuate pressure overload-induced left ventricular hypertrophy through its antioxidant function and subsequent Ask1 inhibition [99]. Notably, edaravone reduced perivascular and intermuscular fibrosis and inhibited pressure overload-induced activation of Ask1 and of its downstream kinases (c-Jun *N*-terminal kinase and p38 MAPK). Edaravone attenuated the hypertrophic response even when treatment was started after onset of cardiac hypertrophic response [104].

**Figure 1:**
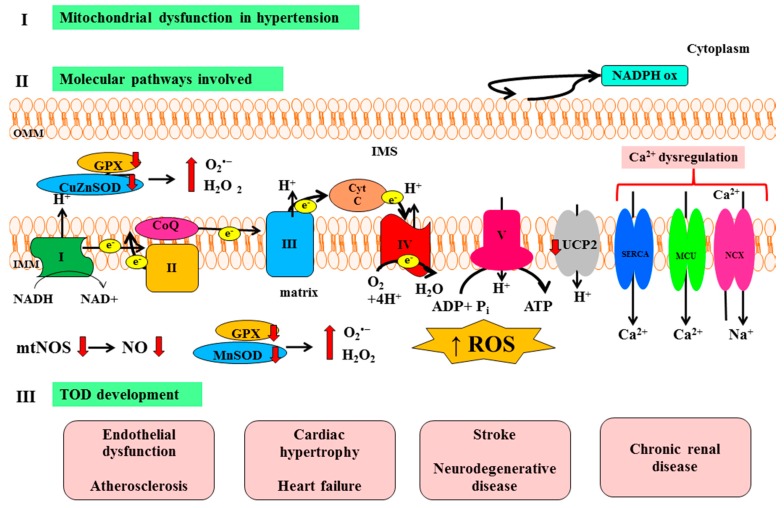
Representation of main molecular pathways linking mitochondrial dysfunction with TOD development in hypertension. NAD: Nicotinamide adenine dinucleotide; NADPH ox: Nicotinamide adenine dinucleotide phosphate oxidase; GPX: Glutathione peroxidase; CuZnSOD: Copper–zinc superoxide dismutase; CytC: Cytochrome C; CoQ: Coenzyme Q; SERCA: Sarco/endoplasmic reticulum Ca^2+^–ATPase; MCU: Mitochondrial Ca^2+^ uniporter; NCX: Na^+^/Ca^2+^ exchanger; ADP: Adenosine diphosphate; ATP: Adenosine triphosphate; mtNOS: Mitochondrial nitric oxide synthase; NO: Nitric oxide; MnSOD: Manganese superoxide dismutase; ROS: Reactive oxygen species; TOD: Target organ damage; OMM: Outer mitochondrial membrane; IMS: Intermembrane space; and IMM: Inner mitochondrial membrane; I: Complex I, NADH dehydrogenase; II: Complex II, succinate dehydrogenase; III: Complex III, cytochrome bc_1_ complex; IV: Complex IV, cytochrome c oxidase; V: Complex V, ATP synthase.

Based on the evidence discussed above, summarized in Figure 1, mitochondrial dysfunction and the consequent oxidative damage emerges as a relevant contributory factor to heart, brain, kidney and vascular damage in hypertension. It is therefore important to continue focusing on targeted mitochondria therapies in order to better understand a major mechanism underlying the pathogenesis of TOD in arterial hypertension.
